# Nutrition attitudes and knowledge in medical students after completion of an integrated nutrition curriculum compared to a dedicated nutrition curriculum: a quasi-experimental study

**DOI:** 10.1186/1472-6920-11-58

**Published:** 2011-08-12

**Authors:** Carolyn O Walsh, Sonja I Ziniel, Helen K Delichatsios, David S Ludwig

**Affiliations:** 1 Boston Combined Residency Program in Pediatrics, Children's Hospital Boston, 300 Longwood Avenue, Boston, MA 02115, USA; 2 Harvard Medical School, 260 Longwood Avenue, Boston, MA 02115, USA; 3 Clinical Research Program, Children's Hospital Boston, 300 Longwood Avenue, Boston, MA 02115, USA; 4 Department of Medicine, Massachusetts General Hospital, 165 Cambridge Street, Boston, MA 02114, USA; 5 Optimal Weight for Life Program, Children's Hospital Boston, 300 Longwood Avenue, Boston, MA 02115, USA

## Abstract

### Background

Nutrition education has presented an ongoing challenge to medical educators. In the 2007-2008 academic year, Harvard Medical School replaced its dedicated Preventive Medicine and Nutrition course with an integrated curriculum. The objective of the current study was to assess the effect of the curriculum change on medical student attitudes and knowledge about nutrition.

### Methods

A survey was administered in a quasi-experimental design to students in the last class of the dedicated curriculum (n = 131) and the first class of the integrated curriculum (n = 135) two years after each class completed the required nutrition course. Main measures were attitude scores based on modified Nutrition in Patient care Survey and satisfaction ratings, performance on a nutrition knowledge test, and demographic variables. Two-tailed t-tests were performed.

### Results

Response rates were 50.4% and 42.2%. There were no differences between the groups in attitude scores from the Nutrition in Patient care Survey (p = 0.43) or knowledge scores (p = 0.63). Students with the integrated curriculum were less satisfied with both the quantity (p < 0.0001) and quality (p = 0.008) of their nutrition education, and were more likely to have completed optional online nutrition training modules (p = 0.0089).

### Conclusions

Medical student attitudes and knowledge about nutrition were not affected by the model of nutrition education they receive, though students in an integrated curriculum may feel their education is inadequate and seek additional training.

## Background

Nutrition plays a major role in the prevention and treatment of many leading causes of disease burden and death worldwide, including cardiovascular and cerebrovascular disease and diabetes mellitus [1-4]. There is a well-documented obesity and overweight epidemic, and nutritional factors such as underweight and micronutrient deficiencies are estimated to affect greater than half of all child deaths worldwide [5-7]. It is therefore imperative that medical schools include nutrition in their curricula. However, it has been difficult for medical schools to meet national guidelines regarding the amount of time spent on nutrition and essential curricular topics [8,9]. The average number of nutrition contact hours was largely unchanged between 1985 (21 hours) and 2008 (19.6 hours), and remains below the 25 hours recommended by the National Academy of Sciences [8,10]. In a 2008 survey of the 127 allopathic medical schools in the United States with graduating classes in the spring of 2009, only 28 of the 105 (27%) that provided information about nutrition contact hours met the 25 hour guideline, down from 40 of 106 (38%) in a corresponding 2004 survey [10,11]. Several studies have shown that the vast majority of medical students and incoming interns are dissatisfied with their medical nutrition education and feel unprepared to counsel patients on nutritional topics [12-14].

Medical nutrition curricula can generally be divided into those with a dedicated nutrition course and those with nutrition content integrated throughout other courses. In the 2008 survey of U.S. medical schools, 26/109 (24%) responding schools had a dedicated curriculum, a decrease from 32/106 (30%) in 2004 [10,11]. One survey of fourth-year students in ten medical schools showed an effect of the type of curriculum on nutrition knowledge, with percent correct on a nutrition knowledge test significantly higher in those with a dedicated, required course than in those with an integrated curriculum [12].

The Preventive Medicine and Nutrition (PMN) course at Harvard Medical School (HMS) has previously been described as an example of a dedicated course. This course contained 28 contact hours over 14 weeks, with each week consisting of a 45-minute lecture and a 90-minute small group exercise, such as problem-based learning, debates, and self-assessment exercises. A pre- and post-test of second-year medical students taking PMN showed an increased confidence in the ability to assess and counsel patients about diet and exercise [15]. During a curriculum revision for the class of 2010, PMN was replaced by an integrated curriculum, with some content presented in a three half-day series called Introduction to Clinical Nutrition (ICN), with lectures and small-group activities, and the rest distributed throughout organ system-based courses. Both courses introduced similar thematic material including macronutrients and micronutrients, obesity, dietary assessment and counseling, amongst other topics.

The objective of the current study was to determine the effect of this curriculum change on HMS students' attitudes and knowledge about nutrition. It was hypothesized that, because nutrition material presented outside of dedicated courses is often not identified as such [16], the ICN students would be less satisfied with their nutrition education than the PMN students. Because of prior studies showing that lower satisfaction with nutrition education is associated with lower knowledge scores [12] and lower nutrition proficiency ratings [17], it was further hypothesized that ICN students would rate lower than PMN students on scales of attitudes toward clinical nutrition and of nutrition knowledge.

## Methods

### Study design and population

The study was a quasi-experimental survey with two groups. Members of both groups were recruited due to enrollment in required nutrition coursework during their second year at HMS. The inclusion criterion for the first group was enrollment in PMN course in the 2006-2007 academic year. The criterion for the second group was enrollment in ICN in 2007-2008, the first year in which this replaced PMN as the required nutrition course. There were no exclusion criteria.

### Ethics

This study received exempt status from the Committee on Human Studies of the Harvard Medical School Office for Research Subject Protection and was performed in accordance with the ethical standards of that organization. The requirement for signed written consent was waived, as consent was implied by participants completing and submitting the survey.

### Survey design

This was a mixed-mode survey with initial web administration followed by a mailed survey for nonresponders. The survey was divided into three sections: attitudes, knowledge, and demographics. The entire survey, containing a total of 60 questions, is available as an additional file (see Additional File 1- Nutrition Education Survey).

#### Attitudes

The attitudes section of the survey contained 30 questions. The first 22 questions came from the existing Nutrition in Patient care Survey (NIPS) [18], which was systematically developed to measure attitudes about the role of nutrition in patient care. With the goal of limiting total survey length so as to maximize response rate, three of the five previously defined NIPS subscales were included: "Nutrition in routine care" (8 items), "Physician-patient relationship" (8 items), and "Physician efficacy" (6 items). All NIPS items were rated on a 5-point Likert scale. Questions 23-29, also rated on a 5-point Likert scale, asked students to rate their satisfaction with the quantity and quality of their medical school nutrition education (2 items) and the extent to which they agree with statements about potential curricular improvements. Question 30 was an open-ended request for ideas to improve the curriculum.

#### Knowledge

The knowledge section contained 21 multiple-choice questions, taken from the online curriculum Nutrition in Medicine (NiM), designed by nutrition faculty at the University of North Carolina for widespread use by medical students [19-21]. Use of the site requires registration and a password from a medical school faculty member. An announcement about the availability of these modules was made during the HMS nutrition course each year. HMS students had access to, but were not required to complete, 24 modules, each followed by a post-test. The 24 post-tests contained 390 questions in total, which were reduced to 87 by taking a random sample of up to four questions from each module. Those 87 questions were reduced to 22 using a discrimination index (DI) derived from data from all online users of the NiM website, who come from more than 200 medical schools worldwide. Individuals were placed into quartiles based on the total percent correct on all 390 items. For each item, the DI was calculated to determine the ability of that item to distinguish overall high performers from low performers:

DI ranges from -1 to +1, with +1 being perfect discrimination between high and low performers, 0 being no discrimination, and -1 being reversed discrimination. A DI cutoff of 0.5 was used, resulting in the 22-item survey component. (One question, whose answer depended upon information not available in the survey, was excluded). As an additional measure of nutrition knowledge, we compared HMS scores on the nutrition section of the Step 1 United States Medical Licensing Examination (USMLE) in 2007 and 2008. There is substantial, but not complete, overlap between students who took the exam in those years and the students who met inclusion criteria for the survey based on nutrition course enrollment.

#### Demographics

The demographics section contained nine questions about potential confounding factors: age, gender, race, ethnicity, height and weight, prior nutrition experience, percentage of NiM modules previously completed, and intended medical specialty.

### Survey administration

The survey was administered to each group two years after their second year of medical school (Spring 2009 for PMN students and Spring 2010 for ICN students). During each administration, the survey was available online for 3 weeks via the HMS intranet site MyCourses, with invitation emails sent weekly. Survey results were anonymous. After completing the survey, participants could contact the study staff in a manner unlinked to their responses to receive a $5 coffee shop gift card. Students who did not report completion of the online survey received a paper copy of the survey in their school mailboxes.

### Statistics

#### Detectable effect

With a response rate of around 50%, power of 0.8 and alpha level of 0.05, detectable effects for the survey were 0.5 for attitudes (18) and 0.225 for knowledge.

#### Atittudes analysis

Five NIPS attitude items were included as validation items stated negatively rather than positively to control for possible response biases (e.g. "Nutrition counseling is *not *an effective use of my professional time"), and were "reverse-scored" prior to analysis (1 changed to 5, etc). Responses were summed for each subscale, with possible scores ranging from 8-40 in the scales with 8 items and from 6-30 in the scale with 6 items. We created a total NIPS attitude score by summing the responses of items 1-22. Two-tailed t-tests (alpha = 0.05, unequal variance) were performed to compare the responses of the two groups on each subscale, the total NIPS score, and each of the additional Likert-based items individually.

#### Knowledge analysis

Each knowledge item was marked as correct or incorrect. A two tailed t-test (alpha = 0.05, unequal variance) was performed to compare the percent of questions answered correctly by the two groups. For the USMLE scores, a standardized curve for all US/Canadian nutrition subscores was produced with mean = 0 and SD = 1, and the HMS nutrition subscore was reported as a z-score compared to the national average.

#### Demographics analysis

Two-tailed t-tests (alpha = 0.05, unequal variance) were performed to compare the demographic characteristics measured as continuous variables, and z-tests (alpha = 0.05) to compare those measured as proportions. Within each year, two-tailed t-tests (alpha = 0.05, unequal variance) were also used to examine the effect of various demographic characteristics on knowledge performance.

#### Missing data

For the NIPS attitude questions, missing data were replaced with the neutral value of 3 so as not to introduce directional bias when the responses were summed. The mean percentage of missing answers for a given NIPS question was 0.5% in 2009 (range 0% to 4.5%) and 0.15% in 2010 (range 0% to 1.75%). On the remaining attitude questions, missing data were not replaced, and the number of respondents for that question was decreased. The mean percentage of missing answers for the remaining attitude questions was 0.65% in 2009 (range 0% to 1.5%); there were no missing answers in this section in 2010. For the knowledge questions, a blank response was considered incorrect. The mean percentage of missing answers for the knowledge questions was 0.4% in 2009 (range 0% to 3%) and 0.2% in 0.17% in 2010 (range 0% to 1.5%). Any survey with > 25% of items missing data was completely removed from analysis (n = 2).

## Results

Responses were received from 66 of 131 PMN students (50.4%), and from 59 of 135 ICN students (43.7%). Two ICN surveys had blank responses for > 25% of items and were removed from analysis, for a final response rate of 42.2% for that group. Demographic characteristics of the two groups are shown in Table 1. There were no significant demographic differences between the two groups.

**Table 1:** Demographic characteristics of second-year Harvard Medical students who responded to the survey

	PMN students^a^	ICN students^b^	p-value
Age in years^c^	26.8 (2.3)	26.9 (2.2)	0.85
% Male	43.1	35.1	0.49
% Hispanic	6.25	8.8	0.84
Ethnicity^d ^(%)			
White	62.5	73.7	0.18
Asian	28.1	15.8	0.19
Black/African-American	6.25	7.0	0.88
American Indian/Alaska Native	0	3.5	0.41
Native Hawaiian/Pacific Islander	0	0	-
Other/Blank	6.25	7.0	0.88
Body mass index^c ^(kg/m^2^)	22.8 (3.7)	22.3 (2.5)	0.42
% with prior nutrition training	19.7	14.0	0.55
% planning a career in primary care^e^	40.9	42.1	0.96

^a^PMN = dedicated Preventive Medicine and Nutrition course.

^b^ICN = integrated Introduction to Clinical Nutrition curriculum.

^c^Age and body mass index presented as mean (standard deviation).

^d^Ethnicity may not equal 100% as individuals could select more than 1 category if applicable.

^e^Percent planning a career in primary care calculated as those indicating a chosen field of family medicine, internal medicine, or pediatrics.

### Attitudes

Table 2 summarizes attitude scores. There were no differences between the two groups in NIPS subscores or total score. However, students with the ICN curriculum were significantly less satisfied with both the quantity and quality of their nutrition education than were students with the dedicated PMN course. Additionally, ICN students had a greater desire, of borderline statistical significance, than PMN students for additional curricular time dedicated to nutrition. There were no differences between the two groups when asked about other potential curricular changes (see Table 2).

**Table 2:** Attitude scores

	PMN students^a, b^	ICN students^b, c^	p-value
NIPS scores^d^			
Nutrition in routine care (8-40)^e^	30.9 (5.8)	30.5 (4.8)	0.68
Physician-patient relationship (8-40)^e^	35.3 (2.9)	35.0 (3.2)	0.65
Physician efficacy (6-30)^e^	18.9 (3.6)	18.2 (3.4)	0.30
Total NIPS score (22-110)^e^	85.1 (9.7)	83.8 (8.5)	0.43

Agreement with the following statements: (1-5)^e^			
"I am satisfied with the quantity of my nutrition education"	3.14 (1.2)	2.26 (0.99)	<0.0001
"I am satisfied with the quality of my nutrition education"	2.67 (1.2)	2.12 (1.1)	.008
"My medical school curriculum should have had more time specifically dedicated to the topic of nutrition (independent of organ system-based studies)"	2.95 (1.2)	3.37 (1.1)	0.051
"My medical school curriculum should have had more nutrition content formally integrated into the organ system-based courses"	3.60	3.63	0.88
"My medical school curriculum should have had more online materials available for independent study"	3.17	3.14	0.88
"My medical school curriculum should have included more material relevant to my personal health and well-being"	3.40	3.26	0.52
"My medical school nutrition curriculum should have been more scientifically rigorous"	3.41	3.49	0.70

^a^PMN = dedicated Preventive Medicine and Nutrition course.

^b^Data are presented as mean (standard deviation).

^c^ICN = integrated Introduction to Clinical Nutrition curriculum.

^d^NIPS = Nutrition in Patient care Survey as designed by McGaghie and colleagues (18).

^e^Numbers inside parentheses in designation lines show possible score ranges.

### Knowledge

There was no difference between the two groups in performance on the knowledge component of the survey (p = 0.63). The average scores for the two groups were 69.3% correct for the PMN group (SD: 0.10) and 68.3% correct for the ICN group (SD: 0.13). Knowledge scores were not different between males and females (2009 p = 0.15, 2010 p = 0.25) or between those who either had prior nutrition experience or who had completed at least 25% of the online NiM modules compared with those who had done neither (2009 p = 0.10, 2010 p = 0.37). Intention to enter a primary care field was associated with a higher nutrition knowledge score in 2009 (p = 0.037), but not in 2010 (p = 0.95). There was also no difference in USMLE nutrition scores between those who took the test in 2007 (mean ± SD: 0.7 ± 0.85) and 2008 (mean ± SD: 0.7 ± 0.75).

### Usage of the NiM modules

The percentage of students reporting completion of at least 25% of the voluntary online NiM modules was significantly higher in the ICN group (33%) than in the PMN group (12.1%) (p = 0.0089).

## Discussion

This study examined the effect of a curricular change at HMS on medical student attitudes and knowledge about nutrition, using modifications of previously developed surveys and questionnaires. There were no differences between students with the two curricula in attitudes towards nutrition in patient care, as measured by the NIPS survey, or in nutrition knowledge, as measured by the NiM-based test and USMLE scores. However, students with the integrated ICN curriculum were less satisfied with both the quantity and quality of their nutrition education. Additionally, ICN students were more likely to use the optional online nutrition modules and, with borderline significance, to report greater desire for additional time dedicated to nutrition. These results suggest that a transition to an integrated curriculum does not necessarily have a detrimental effect on attitudes and knowledge about nutrition, although students may seek additional material to supplement what they perceive as inadequate classroom exposure.

These findings may reassure medical educators working to incorporate nutrition into pre-clinical curricula at a time when an increase in time devoted to nutrition is unlikely. They also provide further evidence that, when asked about nutrition education, students tend to consider only teaching that occurred during a session devoted specifically to nutrition [16]. Therefore, it is not surprising that ICN students reported lower satisfaction with the quantity of their nutrition education, given a decrease in dedicated nutrition time from 28 hours over 14 weeks to 9 hours over 3 days. One medical school successfully targeted this issue by orienting students to the integrated curriculum and attaching a logo to all nutrition material throughout the curriculum [22]; other schools employing an integrated curriculum may consider a similar strategy.

Our study has several strengths. This is the first study to test both attitudes and knowledge in one medical school during a curricular transition. Because comparisons were made within the same school, the student population was likely similar from one year to the next, as reflected in our demographic findings. Additionally, the course director and faculty did not change, so differences between the groups would likely not be attributable to differences in teaching style or ability. In addition, time and clinical exposure after the second-year course were controlled for by administering the survey to each group two years after its required nutrition curriculum.

Several study limitations warrant comment. The participation rate was moderate, though comparable to similar studies [14,17], and thus selection bias may be present. Demographic data of non-responders was not available, but it is possible that, compared with responders, non-responders may be less interested in nutrition, less likely to complete the NiM modules, and less likely to enter primary care fields. These factors are likely similar between the two groups. Students were not randomized; instead the quasi-experimental design was used to take advantage of the planned curricular change. Each group was surveyed at only one time point at the end of medical school, so we cannot assess pre- and post-course changes in attitudes and knowledge. The survey was administered to each group two years after completion of the respective curricula; it is possible that unmeasured confounding events occurred during that time. Also, as the two groups were surveyed one year apart from each other, it is possible that historical events in the intervening year affected the attitudes and knowledge of the second group. It is not clear whether the results would generalize to other medical schools. The knowledge section was developed to capitalize on the existing multiple choice questions and DI data of the NiM website. It is possible that we may have found a difference in knowledge scores had we instead developed questions based on the PMN syllabus. Lastly, as this study focused on pre-clinical curricular choices, it did not address the effect of continued nutrition education opportunities during clinical training and practice, the importance of which has been previously described [16] and which has led to the recent development of the NiM program, Nutrition Education for Practicing Physicians [23].

## Conclusions

Appropriate nutrition education for medical professionals is likely to have a positive impact on patient care and health outcomes. Interventions designed to increase physician nutrition knowledge can increase the rates at which physicians discuss nutrition and recommend specific dietary interventions with patients [24], and patients who are advised by a physician to make lifestyle modifications such as dietary changes are more likely to do so than those who receive no such advice [25]. Based on the current results, while students may prefer a dedicated nutrition course and seek supplemental training when nutrition content is integrated, students emerge from these two methods of nutrition education with equal attitudes toward clinical nutrition, suggesting equal likelihood of counseling patients, and with similar nutrition knowledge bases. Future work should determine exactly how likely these students are to counsel patients and which educational techniques lead to increased incorporation of nutrition knowledge into clinical care.

### Ethical approval

This study received exempt status from the Committee on Human Studies of the Harvard Medical School Office for Research Subject Protection.

## Competing interests

The authors declare that they have no competing interests.

## Authors' contributions

Author contributions: COW and DSL designed and implemented the study. SIZ advised in survey design and statistical analysis. All authors contributed to data interpretation and manuscript writing, and have read and approved the final manuscript.

## Pre-publication history

The pre-publication history for this paper can be accessed here:

http://www.biomedcentral.com/1472-6920/11/58/prepub

## Supplementary Material

Additional file 1 **Nutrition Education Survey**. Complete survey instrument. Click here for file
