# Supplementary material for: Nutrition attitudes and knowledge in medical students after completion of an integrated nutrition curriculum compared to a dedicated nutrition curriculum: a quasi-experimental study
Source: BMC Med Educ. 2011 Aug 12;11:58. doi: 10.1186/1472-6920-11-58 (PMC3173384; doi:10.1186/1472-6920-11-58)
Supplement: Additional file 1 — Nutrition Education Survey. Complete survey instrument. [file 1472-6920-11-58-S1.DOC]

**Nutrition Education Survey**

**SECTION 1- ATTITUDES**

**Directions:** Each statement below finishes the sentence “In general, I believe that . . .” Circle the response that is closest to your opinion about each statement.

**1= Strongly Disagree, 5= Strongly Agree**

| *1. Preventive health care is *boring.* | 1 2 3 4 5 |
| --- | --- |
| 2. Nutrition counseling should be part of routine care by all physicians, regardless of specialty. | 1 2 3 4 5 |
| 3. Nutritional assessment and counseling should be included in any routine appointment, just like diagnosis and treatment. | 1 2 3 4 5 |
| *4. Nutrition counseling is *not* an effective use of my professional time. | 1 2 3 4 5 |
| *5. Individual physicians have *little* impact on a patient’s ability to lose weight. | 1 2 3 4 5 |
| 6. I have an obligation to improve the health of my patients including discussing nutrition with them. | 1 2 3 4 5 |
| 7. All physicians, regardless of specialty, should counsel high-risk patients about dietary change. | 1 2 3 4 5 |
| *8. It is *not* worth the time to counsel patients with poor dietary patterns about nutrition. | 1 2 3 4 5 |
| 9. Patient motivation is essential to achieving dietary change. | 1 2 3 4 5 |
| 10. Most obese patients want to lose weight but feel frustrated and confused about how to do it. | 1 2 3 4 5 |
| 11. Patients need good-tasting alternatives in order to change their eating patterns. | 1 2 3 4 5 |
| 12. A change toward a healthier lifestyle is important at any stage of life. | 1 2 3 4 5 |
| 13. Most physicians are *not* adequately trained to discuss nutrition with patients. | 1 2 3 4 5 |
| 14. Patients need specific instructions about how to change their eating behavior. | 1 2 3 4 5 |
| 15. Specific advice about how to make dietary changes could help some patients improve their eating habits. | 1 2 3 4 5 |
| 16. Patients need ongoing counseling following my initial instruction to maintain behavior changes consistent with a healthier diet. | 1 2 3 4 5 |
| 17. Most patients will try to change their lifestyle if I advise them to do so. | 1 2 3 4 5 |
| 18. Physicians can have an effect on a patient’s dietary behavior if they take the time to discuss the problem. | 1 2 3 4 5 |
| *19. For most patients, health education does *little* to promote adherence to a healthy lifestyle. | 1 2 3 4 5 |
| 20. After receiving nutrition counseling, patients with poor eating habits will make major changes in their eating behavior. | 1 2 3 4 5 |
| 21. My patient-education efforts will be effective in increasing patients’ compliance with nutritional recommendations. | 1 2 3 4 5 |
| 22. After receiving nutrition counseling, patients with poor eating patterns will make moderate changes in their eating behavior. | 1 2 3 4 5 |

*Items 1, 4, 5, 8, and 19 were reverse-scored (1 changed to 5, etc) prior to analysis

**DIRECTIONS:** The following questions measure your opinions about your nutrition education in medical school. Please indicate the extent to which you agree with the following statements.

**1= Strongly Disagree, 5= Strongly Agree**

| 23. I am satisfied with the quantity of my nutrition education. | 1 2 3 4 5 |
| --- | --- |
| 24. I am satisfied with the quality of my nutrition education. | 1 2 3 4 5 |
| 25. My medical school nutrition curriculum should have had more time specifically dedicated to the topic of nutrition (independent of organ system-based studies). | 1 2 3 4 5 |
| 26. My medical school nutrition curriculum should have had more nutrition content formally integrated into the organ system-based courses. | 1 2 3 4 5 |
| 27. My medical school nutrition curriculum should have included more online materials available for independent study. | 1 2 3 4 5 |
| 28. My medical school nutrition curriculum should have included more material relevant to my personal health and well-being. | 1 2 3 4 5 |
| 29. My medical school nutrition curriculum should have been more scientifically rigorous. | 1 2 3 4 5 |

30. Other ideas for improving the nutrition curriculum:

**SECTION 2- KNOWLEDGE**

**Directions:** For the following questions, please place a check mark next to the one best answer.

1. It has been proposed that a low omega-3 fatty acid intake increases risk of some cancers. A study investigated this possible link by measuring adipose tissue composition (a marker for past omega-3 fatty acid intake) in newly diagnosed patients. What additional information is needed to make this a useful comparison?

___ a. Time lapse since diagnosis

___ b. Information on family cancer risk

___ c. Adipose tissue composition of patients without cancer

___ d. Endoscopy results showing the absence of colon cancer

___ e. Dietary assessment of current omega-3 and omega-6 fatty acid intake

2. A prospective study of pack-a-day smokers assessed customary food intakes and carried out annual diagnostic tests to detect newly formed lung cancers. After ten years, fewer of the participants with high fruit and vegetable intakes had new cancer than those with low consumption. What do you need to look for in the report to assess the validity of the study?

­­­___ a. Was fat intake monitored?

___ b. Were cancer patients matched for sex and age?

___ c. Was cigarette exposure measured with a biomarker?

___ d. Was dietary information available from most of the participants?

___ e. Were appropriate markers used to exclude people with genetic cancer risk?

3. A 66-year-old obese black male comes to see you about his Type 2 diabetes. In looking over his chart, you see that since his last visit he has developed mild hypertension. You check the blood pressure on several more occasions and it is still high. In order to decide how to treat the hypertension, you ask the patient if he is willing to go on a diet to restrict:

___ a. calcium

___ b. potassium chloride

___ c. sodium chloride

___ d. magnesium

___ e. folate

4. You find that a patient has a prolonged bleeding time after she was started on an antibiotic. Which foods might have prevented this side effect of the antibiotic?

___ a. cold water fish

___ b. beef and pork

___ c. dairy products and eggs

___ d. oranges, grapefruit, and tomatoes

___ e. cooked greens and green vegetables

5. You discuss the importance of diet with a 12-year-old with diabetes. If the patient eats more than she is supposed to at dinner one night, compared to her usual blood sugars, you would expect her blood glucose concentration to be:

___ a. lower in the morning

___ b. higher at bedtime

___ c. higher after she exercises

___ d. lower at bedtime

___ e. lower in the middle of the night

6. The formula of an infant with maple syrup urine disease should limit the quantity of which amino acids?

___ a. Threonine, methionine, and cysteine

___ b. Proline and arginine

___ c. Tyrosine and phenylalanine

___ d. Alanine, glycine, and serine

___ e. Isoleucine, leucine, and valine

7. How is the brain’s need for energy met after an overnight fast?

___ a. glycogen in the brain is mobilized

___ b. glucose is transferred from the liver

___ c. fatty acids are mobilized from adipose tissue

___ d. liver generates ketone bodies

___ e. lactate is transferred from muscles

8. How does low bile secretion impact nutrient disposition?

___ a. lack of enterokinase activation by bile acids slows protein digestion

___ b. diminished protease activity will limit bioavailability of biotin

___ c. reduced biliary losses will cause excessive zinc storage

___ d. impaired micelle formation will limit absorption of vitamin D

___ e. low enzyme secretion with bile slows oligopeptide digestion

9. Your patient is a 12-year-old boy who is 150 cm (59 in) tall and weighs 45 kg (100 lbs). He has recently gained 2.7 kg (6 lb). You suspect that:

___ a. the boy must be under active since he is putting on extra weight

___ b. he is likely retaining water

___ c. he could be showing early signs of an eating disorder

___ d. his body is preparing for the growth spurt of adolescence

___ e. he probably has cystic fibrosis

10. The sudden drop in progesterone post-partum stimulates:

___ a. The contraction of the uterus

___ b. The onset of menses

___ c. Mobilization of fat stores

___ d. The onset of copious milk secretion

___ e. The let-down response

11. An important component of human milk that is conditionally essential in young infants is:

___ a. Cholesterol

___ b. α-linolenic acid

___ c. Lactose

___ d. Lactoferrin

___ e. Docosahexaenoic acid

12. When evaluating a maternal diet, it is important to assess intake of energy, protein, and micronutrients because:

___ a. Low protein intake in 3^rd^ trimester increases risk of preeclampsia

___ b. High energy intake in 2^nd^ & 3^rd^ trimesters may cause microsomia

___ c. Deficient glucose intake in all trimesters may cause macrosomia

___ d. Low iron intake in 3^rd^ trimester may slow brain development

___ e. High protein intake in 1^st^ trimester impairs fetal renal function

13. You’ve been asked to talk to a middle school health education class about common health problems in the US. You decide to focus on obesity as a component of many medical problems. You tell the class that the prevalence of obesity in the US is about:

___ a. 20% of adults and children

___ b. 60% of adults and children

___ c. 15% of adults

___ d. 30% of adults

___ e. 50% of adults

14. You have a patient who exercises several hours per day, 3-5 times per week. He is concerned about getting enough potassium, but dislikes bananas. What other food sources can you suggest?

___ a. pretzels

___ b. tomatoes

___ c. rice cakes

___ d. eggs

___ e. jello

15. A 50-year-old man has fallen off a scaffold and suffered severe head injuries and multiple bone fractures. He has been unconscious for ten days. Which is the most important reason to provide nutrition support for this patient?

___ a. Nutrition support is needed to counter side effects of antibiotics on the gut

___ b. To decrease the morbidity associated with malnutrition

___ c. He must regain the muscle mass lost during ten days of immobilization

___ d. To avoid trace mineral and essential fatty acid deficiency

___ e. The fatty acids released from adipose tissue during fasting will cause fatty liver

16. In a patient who has been maintained for four days on only intravenous fluids following a right hemicolectomy, increased output of urinary urea is likely to be:

___ a. due to increased input of intravenous fluids

___ b. due to increased catabolism of body proteins

___ c. a sign of early hepatic failure

___ d. due to post-operative release of anti-diuretic protein hormone

___ e. due to post-operative release of adrenal steroids

17. After food is ingested orally or enterally, the absorbed fats are first transported:

___ a. To the liver via enterohepatic circulation

___ b. To the lymphatic system via chylomicrons

___ c. To the circulatory system as droplets without apoproteins

___ d. To the brain via lipoproteins

___ e. To the lungs via apoB

18. A discrepancy in the effectiveness of a synthetic nutrient and a natural one is often due to differences in:

___ a. bioavailability

___ b. packaging

___ c. safety

___ d. side effects

___ e. manufacturing

19. A 50-year-old woman takes a “healthy bones” dietary supplement she found on the internet that contains synthetic chemicals with structures similar to glucosamine and condroitin. You are familiar with research about glucosamine and condroitin, so you can make the following assumption:

___ a. The new supplement would achieve the same results because of similar chemical structures

___ b. The new supplement would not achieve the same result because the chemical structures are not identical

___ c. The new supplement would have the same action as long as it is easily absorbed

___ d. You can assume the new supplement is easily absorbed because it is in synthetic form

___ e. You cannot make any assumptions about the new supplement

20. A teenager who is on a “no-fat” diet is at risk for deficiency of which vitamin?

___ a. Vitamin E

___ b. Vitamin C

___ c. Folate

___ d. Riboflavin

___ e. Pantothenate

21. A 65-year-old male newly diagnosed with Alzheimer’s disease and his wife visit your clinic. He is reluctant to take medication that may slow the progression of the disease and would rather take ginkgo biloba as a natural alternative. You are most likely to find reliable information in which location?

___ a. DRI publications

___ b. The Dietary Guidelines

___ c. A literature search

___ d. Alzheimer’s website

___ e. Systematic review

**SECTION 3- DEMOGRAPHICS**

**Directions:** Please complete the following demographic questions.

1. How old are you? _________ years old

2. What is your gender? ____Male ____ Female

3. Are you Hispanic or Latino? ____Hispanic or Latino ____Not Hispanic or Latino

4. What is your race? (check all that apply)

___ American Indian/Alaska Native

___ Asian

___ Black/African-American

___ Native Hawaiian/Pacific Islander

___ White

___ Unknown

___ Other (please explain): _________________________________________

5. What is your height? _____ inches

6. What is your weight? ______ lbs

7. Did you have any nutrition training prior to medical school?

___Yes: ___ College coursework (How many courses?___ 1, ___2, ___3+)

___ RD

___ PhD

___ Physical trainer

___ Other (please explain): _______________________________________

___No

8. Which of the following best represents the percentage of modules you have ever completed in the online curriculum Nutrition in Medicine?

___ 0% ___ 25% ___ 50% ___ 75% ___ 100%

9. What is your intended specialty?

___ Anesthesiology

___ Dermatology

___ Emergency Medicine

___ Family Medicine

___ Internal Medicine

___ Neurology

___ Obstetrics and gynecology

___ Ophthalmology

___ Pathology

___ Pediatrics

___ Psychiatry

___ Radiology

___ Surgery: ___ General (If General Surgery, please respond: I am considering a career in

bariatric surgery ___ Yes ___ No)

___ Subspecialty

___ Don’t know

___ Other (please explain): ________________________________________
